# Actinium-225 as an example for monitoring of internal exposure of occupational intakes of radionuclides in face of new nuclear-medical applications for short-lived alpha emitting particles

**DOI:** 10.1007/s00411-024-01081-4

**Published:** 2024-07-20

**Authors:** Sven Hartmann, Kerstin Taubner, Tobias Vogt, Oliver Meisenberg, Uwe-Karsten Schkade, Christian Steyer, Marian Meckel, Christian Kesenheimer

**Affiliations:** 1 Federal Office for Radiation Protection, Medical and Occupational Radiation Protection, Incorporation Monitoring MB 5, Berlin, Germany; 2 Federal Office for Radiation Protection, Environmental Radioactivity, Dosimetry and Spectrometry UR 5, Berlin, Germany; 3 ITM Medical Isotopes GmbH, Munich, Germany

**Keywords:** Actinium-225, Monitoring of internal exposure, Radiation protection, Alpha spectrometry

## Abstract

Monitoring of internal exposure to short-lived alpha-emitting radionuclides such as actinium-225 (^225^Ac), which are becoming increasingly important in nuclear medicine, plays an important role in the radiation protection of occupationally exposed persons. After having tested gamma spectrometry, liquid scintillation counting and alpha spectrometry for monitoring of internal exposure, the focus of the present study was on solid phase extraction of ^225^Ac from urine in combination with alpha spectrometry. The development of the method was based on recent findings from the literature on this topic. The method was used in a pilot phase to monitor internal exposure of four workers who were directly or indirectly involved in the manufacture and/or use of ^225^Ac. The monitoring protocol allowed a relatively short 24-hour urine sample analysis with excellent recovery of the internal standard, but it did not allow for a detection limit of less than 1 mBq nor a sufficient yield of ^225^Ac. Based on these results it is concluded that an in vitro excretion analysis alone is not appropriate for monitoring internal exposure to ^225^Ac. Instead, different radiation monitoring techniques have to be combined to ensure the radiation protection of employees.

## Introduction

In 2021, Sung et al. (2021) reported statistics on cancer, which is one of the top reasons for premature mortality (age 0–69) in most parts of the world, especially Europe and North America, thus making it a great challenge for human health. The treatment of cancer in earlier years mostly depended on cytotoxic chemotherapies, whereas in the last decades new techniques were developed (Zugazagoitia et al. 2016). One of those techniques is targeted alpha therapy (TAT), which is based on linking a cancer-cell specific molecule to a chelated alpha-emitting radioactive nuclide (Kim and Brechbiel 2012; Morgenstern et al. 2018). The resulting active compound docks on specific areas of the cancer cell, with the alpha-particles emitted by the compound destroying essential parts of the cancer cell with minimal collateral damage. In some cases, this approach resulted in very promising treatments (Kim and Brechbiel 2012; Morgenstern et al. 2018). Right now, there are eight alpha-emitting radionuclides in focus of such radiopharmaceuticals (Eychenne et al. 2021), of which one is actinium-225 (^225^Ac). ^225^Ac has a physical half-life of about 10 days and, due to five short-lived daughter nuclides of which three emit alpha particles, a high dose coefficient of 1.8·10^− 6^ Sv/Bq (for inhalation, solubility class M corresponding to medium solubility, activity median aerodynamic diameter (AMAD) 5 μm) (ICRP 2022).

In accordance with the German Radiation Protection Act, German companies working with ^225^Ac must, under certain conditions, monitor employees who handle ^225^Ac (occupationally exposed persons) (ISO 20553 2006; Radiation Protection Act 2022). Monitoring for internal exposure can take place in various ways, e.g. by whole-body measurement using gamma spectrometry (in vivo) or by excretion analysis (in vitro) (ISO 20553 2006). Given the biokinetic data of ^225^Ac as provided by the International Commission on Radiological Protection (ICRP) (for inhalation, type M as the default for actinium (ICRP 2022), a monitoring interval of ≤ 7 days for in vitro analyses and of ≤ 30 days for in vivo analyses would have to be used for employees to satisfy Eqs. 3 and 4 of ISO 20553 (2006). For in vitro analyses, this would require measurements every week (i.e., 52 per year) and one would then have to detect 1/52 of the yearly dose limit of 1 mSv per measurement. This can be achieved with in vitro analyses, given the fact that the detection limit of e.g. alpha spectrometry is lower than that of gamma spectrometry (Jia and Jia 2012). However, to date there is no method available for the monitoring of internal exposure of ^225^Ac using e.g. urine samples. However, recently it has been shown that ^225^Ac can be quantitively extracted from urine using a Y-imprinted resin for solid-phase extraction (Cusnir et al. 2022). This method served as the basis for the present study where this method was modified and applied in the context of internal monitoring. For this purpose, a collaboration with Isotope Technologies Munich (ITM) was established, because this company works on ^225^Ac radiopharmaceuticals, amongst other things. Due to the high activities to be handled (MBq to GBq) by ITM in combination with the high dose coefficient of ^225^Ac, a potential effective dose of more than 1 mSv per calendar to ITM employees cannot be ruled out. This would require regular monitoring of internal exposure of the employees (ISO 20553 2006). As mentioned above, employees would have to be measured or, in case of in vitro monitoring, would have to collect one 24-hour urine sample per week (a total of 52 samples per year, leading to 1/52 mSv per sample to be detected). This would mean an activity to be determined in the order of 1 mBq per sample (calculated using OIR Data Viewer v. 5.27.9.21 (ICRP 2022). Therefore, any method would have to fulfil the following requirements:

1. A short analysis time, as samples must be processed weekly,
2. the detection limit must be lower than the activity to be detected of 1 mBq,
3. the chemical yield of ^225^Ac or, in case of alpha spectrometry, of the internal standard should be > 50% (Vogl 1992),
4. the chemical yield of the internal standard and ^225^Ac should be in a similar range, and.
5. for routine application of the method, only commercially available materials and standard radiochemistry laboratory equipment is preferred.

In order to develop such a method for the radiation protection of individuals with potential occupational intake of ^225^Ac, different methods were explored. The focus was on testing a promising method for the practical monitoring of internal exposures with four probands, in order to explore the applicability and practicability of an in vitro excretion analysis method in the near future.

## Materials and methods

### Chemicals and radioactive substances

If not stated otherwise, all chemicals were from Chemsolute^®^ (Th. Geyer GmbH & Co. KG, Germany) or Supelco^®^ (Merck KGaA, Germany) in the grade p.a. Radioactive substances were purchased from Eckert & Ziegler Nuclitec GmbH (Germany) or Physikalisch-Technische Bundesanstalt (PTB, Germany); their activities were traceable to national standards. ^225^Ac was provided as ^225^Ac-chloride solution in 0.04 M HCl by ITM. Deionized water (dH_2_O) was created using an in-house deionization system. Synthetic urine was purchased from Synthetic Urine e.K. (Germany).

### Calculation of radionuclide activity, yield, effective dose and detection limit

The ^225^Ac yield (η_Ac−225_) from in vitro measurements (i.e. radiochemical measurements in course of the excretion analysis) was calculated using Eq. (1):

1 $$\:{\eta\:}_{\text{Ac}\text{-225}}=\:\frac{\frac{\varphi\:\:\left({R}_{\text{b}}-{R}_{0}\right)\:}{\epsilon\:\:}}{{a}_{\text{Ac-225}}}$$

Where ϕ is the calibration factor in Bq/s, *R*_b_ is the counting rate of the sample in 1/s, *R*_0_ is the counting rate of a blank sample in 1/s, ε is the emission probability of ^225^Ac, and a_Ac−225_ is the added activity of ^225^Ac in Bq.

The yield of the tracer used (^243^Am, also referred to as “internal standard”) was calculated analogous to Eq. (1).

The effective dose was calculated according to ISO 20553 (2006). The detection limit was calculated according to DIN EN ISO 11929 (2021).

All uncertainties are presented with a coverage factor of *k* = 1.

### Gamma spectrometry

Samples consisted of 200 mL synthetic urine spiked with ^225^Ac solution. The samples were filled in 250 mL polyethylene flasks with a fill height of 8 cm. All samples were measured with a p-type pure Germanium (ultra-low-level configuration) detector, Model GEM-FX 7030-S relative efficiency 32.3% (ORTEC, USA). The ^225^Ac activity concentration was determined via the gamma line of its daughter nuclide ^221^Fr at the gamma energy of 218.2 keV, whereby radioactive equilibrium between ^225^Ac and its short-lived daughter nuclides was assumed.

### Liquid scintillation counting measurements

For LSC measurements, LSC vials were filled to 8 mL with dH_2_O, spiked with a ^225^Ac solution, and 12 mL of a scintillation cocktail Ultima Gold™ AB (PerkinElmer Inc., USA) were added. The samples were measured using Hidex 300SL (Hidex Oy, Finland) for 12,000 s using an alpha/beta discrimination method from Hidex Oy, Finland, with an alpha energy region from 2.7 to 1,800 keV and a beta energy region from 0.1 to 1,800 keV. For the background, samples without the addition of ^225^Ac were measured simultaneously. The analysis was performed using MikroWin 300SL v.5.53 software (Hidex Oy, Finland).

### 24-hour urine samples

For the pilot-phase 24-hour urine samples were collected by the probands. The collection was done using 2.7 L PET flasks, and should involve the morning urine from the starting day and the day after. Then the samples were tightly closed, wrapped in cellulose and plastic bag and shipped to the laboratory within 36 h.

### Excretion analysis

The excretion analysis was based on recent work from Cusnir et al. (2022). The finally developed method is presented below.

#### Sample preparation

Urine samples were divided into 500 mL aliquots. These aliquots were acidified with 5 mL HNO_3_ (14.4 M).

#### Coprecipitation (step 1)

To the 500 mL aliquots of urine samples a ^243^Am tracer solution including 50–250 mBq sample activity was added. If required for pre-trials and QA samples (quality assuring (QA) samples during the pilot-phase, provided by ITM) ^225^Ac was also added in varying activity ranges. The samples were then stirred and heated near boiling point until the urine cleared up. The hot plate was then switched off and 5 mL Ca(NO_3_)_2_ solution (20 mg/mL) was added, following the addition of 2 mL H_3_PO_4_ (6 M). To induce the coprecipitation an NaOH solution (32% w/v) was added until pH 8–9. The samples were stirred for 30 min at 70 °C and afterwards the precipitate was allowed to settle down for 2–3 h.

#### Digestion of organic matter (step 2)

The precipitate was decantated from the urine and transferred to a centrifuge tube with dH_2_O. The mix was centrifuged for 5 min at 4000 rpm (rotations per minute) and room temperature. The supernatant was discarded, the precipitate solved in 5 mL HNO_3_ (14.4 M) and 1 mL H_2_O_2_ (30% v/v) and transferred to a microwave tube. Organic matter was digested using a microwave system for 20 min at 40 bar and 180 °C. The resulting solution was transferred to a beaker and filled up to 20 mL with dH_2_O.

#### Solid-phase extraction (step 3)

For solid-phase extraction a vacuum box system (Triskem International, France) with diglycolamide (DGA) branched resin prepacked cartridges (2 mL bed volume, 50–100 μm particle size, Triskem International, France) were used. The cartridges were conditioned using 5 mL HNO_3_ (4 M) twice. Flow rate was adjusted to 1–2 mL/min. The 20 mL sample solution was loaded onto the cartridges and the flow through was discarded. For the washing step 5 mL HNO_3_ (4 M) were used twice and flow throughs were discarded. For elution 15 mL HCl (0.25 M) was used and the eluate was collected in a beaker.

#### Electrodeposition (step 4)

The electrodeposition protocol was based on previous work (Bajo and Eikenberg 1999). For electrodeposition, stainless steel discs were placed in electrolysis cells. To the 15 mL sample solution 0.6 mL NaHSO_4_ (1 M) and 0.4 mL H_2_SO_4_ (18.2 M) were added. The solution was slowly heated on a sand bath (increasing temperature) till dry. Afterwards the sample was calcinated for 30–60 min. The beaker was removed from the sand bath and the residue was dissolved in 0.3 mL H_2_SO_4_ (18.2 M). The beaker was again placed on a sand bath, heated until fumes formed and removed again from the sand bath. When cool, the residue was dissolved in 5 mL dH_2_O and transferred to an electrolysis cell. The beaker was rinsed twice with 5 mL dH_2_O and the solution was also added to the electrolysis cell. The pH was adjusted to 2.4 with H_2_SO_4_ (1.5 M) and NH_3_ solution (13.4 M). Electrolysis was performed at 1.2 A for 75 min. One minute before the end 1 mL NH_3_ solution (13.4 M) was added to the electrolysis cell. After electrolysis the stainless-steel disc was washed with dH_2_O and dried on air.

#### Alpha spectrometry (step 5)

For alpha spectrometry an Alpha Analyst™ type 7210 alpha spectrometer (Mirion Technologies (Canberra) GmbH, Germany) with PIPS^®^ detectors (Mirion Technologies (Canberra) GmbH, Germany) were used. The efficiency calibration was traceable to a national standard. The samples were measured for 20,000 s under low pressure. For background, untreated stainless-steel disks were measured for 300,000 s under low pressure. For analysis Genie™ 2000 v3.3 software (Mirion Technologies (Canberra) GmbH, Germany) was used.

## Results

In order to develop a practical analytic method for the excretion analysis of ^225^Ac from 24-hour urine samples, different preliminary tests were conducted. First, the three methods alpha spectrometry (AS), liquid scintillation counting (LSC) and gamma spectrometry (GS) were compared. Afterwards, the most promising method in terms of yield, detection limit and processing time was applied for a pilot-phase with real 24-h urine samples. For method selection (i.e. AS vs. LSC vs. GS) a pure ^225^Ac-chloride (AcCl_3_) solution was kindly provided by ITM, typically 100 µL HCl (0.04 M) with 100 kBq of activity, which was further diluted for the tests.

### Gamma spectrometry and LSC

#### Gamma spectrometry

Gamma spectrometry was tested by preparing samples consisting of 200 mL synthetic urine with different ^225^Ac activities in the range of 9 to 20 Bq. This method allowed a direct measurement of samples without any previous sample preparation. Six samples (GS1-GS6) were measured on a pure Germanium detector in polyethylene flasks with a fill height of 8 cm. The samples consisted of 200 mL synthetic urine with ^225^Ac. Added ^225^Ac activity values with resulting recovery rates, measurement times and detection limits (for a daily urine excretion of 1.4 L) were: 10.5 Bq, 80.0% ± 5.7%, 315,000 s, 1.4 Bq for GS1; 17.5 Bq, 103.5% ± 8.1%, 80,000 s, 4.2 Bq for GS2; 17.7 Bq, 77.8% ± 7.3%, 160,000 s, 6.2 Bq for GS3; 8.8 Bq, 81.2% ± 7.8%, 160,000 s, 6.4 Bq for GS4; 9.4 Bq, 95.3% ± 7.5%, 320,000 s, 4.9 Bq for GS5; and 18.7 Bq, 95.6% ± 8.0%, 320,000 s, 6.3 Bq for GS6. Uncertainties were determined regarding DIN 1319-1 (1995). For all samples the activity was measured once. The samples were measured for 80,000 s to 320,000 s. The results showed that for all six samples measured (GS1 – GS6), the recovery rate was higher than 78%. With a mean value of the six samples of 89 ± 10% the GS proved to be very efficient regarding ^225^Ac recovery. Yet, even with a measurement time of 320,000 s (i.e. ~4 d), the lowest detection limit (for a daily urine excretion of 1.4 L) obtained was 1.4 Bq, which is significantly higher than the required detection limit of less than 1 mBq.

#### LSC measurements

Unlike the GS samples, the LSC samples did not contain synthetic urine. A specific ^225^Ac activity was added to LSC vials containing 8 mL dH_2_O and 12 mL scintillation cocktail (Ultima Gold™ AB). Six samples were prepared (LSC1-LSC6) with ^225^Ac activity in the range of 0.6 to 600 Bq, which were measured for 12,000 s using the alpha/beta discrimination method from Hidex Oy.

For the set ^225^Ac activity values, measured ^225^Ac activity values, yields and detection limits the following results were obtained: 592 Bq, 148 Bq ± 30 Bq, 25%, 4.5 Bq for LSC1; 297.3 Bq, 76.8 Bq ± 15.4 Bq, 26%, 3.2 Bq for LSC2; 146.3 Bq, 39.8 Bq ± 8.0 Bq, 27%, 2.3 Bq for LSC3; 20.7 Bq, 7.9 Bq ± 2.0 Bq, 38%, 1.6 Bq for LSC4; 5.9 Bq, 2.0 Bq ± 0.5 Bq, 34%, 490 mBq for LSC5; and 0.6 Bq, 0.006 Bq ± 0.003 Bq, 1%, 510 mBq for LSC6. The measured value of LSC6 6 mBq was lower than the detection limit of 510 mBq and the yield only 1%. Values given are mean values of two technical replicas for each sample. The uncertainties were determined using the software MikroWin 300SL v.5.53 and the standard deviation of the mean value. The results showed for all samples, except LSC6, reasonable ^225^Ac yields with 25–38%, which is still lower than expected, possibly due to problems with alpha-beta discrimination. In addition, the detection limit was for example about 490 mBq for sample LSC5, which is lower compared to those obtained for the GS samples. However, it was still higher than the required detection limit of 1 mBq. Moreover, for monitoring of internal exposure, the necessary ^225^Ac activity value to be measured would be in an activity range of 1–50 mBq. The only sample somewhat close to that value was LSC6 (set ^225^Ac value 600 mBq), which showed difficulties in the evaluation. Also, further measurements (data not shown) of set ^225^Ac values lower than 2 Bq showed similar results. Therefore, LSC seemed not sufficient and the study was continued with the AS.

### Solid-phase-extraction in combination with alpha spectrometry

Recent work on the solid-phase extraction of ^225^Ac from urine showed one of the first examples for a qualitative and quantitative radiochemical analysis of low ^225^Ac activities (Cusnir et al. 2022). However, these authors used a self-made Y-imprinted resin for solid-phase extraction chromatography, which can make the procedure somewhat challenging for in vitro monitoring by internal exposure laboratories, since the equipment and technical perquisites required may not be available to them. Nonetheless, the present work is based on the protocol and results of Cusnir et al. (2022) with the aim of using only commercially available reagents and materials, making the method easier to be used for a widespread future application of in vitro monitoring of internal exposure to ^225^Ac.

In the present study, the steps described in the Materials and Method section were combined in three different ways, i.e., the complete analysis (steps 1–5), the separation (step 3 to step 5) and the electrodeposition (step 4 to step 5). For the first preliminary tests an AcCl_3_ solution was used. Additionally, ^243^Am was used as a tracer for radiochemical yield determination (Cusnir et al. 2022). Due to a limited supply of AcCl_3_ in the phase of the preliminary tests, the first experiments for each mentioned group (i.e. complete analysis, separation and electrodeposition) could only be conducted once. For the adapted method the corresponding ^225^Ac yields and ^225^Ac/^243^Am yield ratios were: Electrodeposition: 45.3%, 0.8; Separation: 3.4%, 0.1; Complete: 0.01%, 0.1.

For comparison of ^225^Ac yields and ^225^Ac/^243^Am yield ratios, literature values were calculated as mean values from the corresponding Tables: 1 (Electrodeposition), 2 (Separation) and 3 (Complete) (Cusnir et al. 2022), with the uncertainty presented being the standard deviation of the values. The corresponding ^225^Ac yields and ^225^Ac/^243^Am yield ratios were: Electrodeposition: 100.6% ± 1.6%, 1.1; Separation: 68.4% ± 15.5%, 0.9; Complete: 54.9% ± 19.4%, 0.9.

First, the electrodeposition (i.e. steps 4–5) was examined starting with a 0.25 M HCl solution, as this represented the actual elution solution form the previous step 3. 270 mBq ^225^Ac and 210 mBq ^243^Am were added, and the sample was treated according to the literature (Cusnir et al. 2022) by adjusting the pH to 1.9. Electrolysis was carried out at 1.2 A for 75 min. A stainless-steel disc was then measured for 20,000 s in the alpha spectrometer. The results showed a yield of 45% for ^225^Ac, which was about 55% lower than the reported values from Cusnir et al. (2022). However, the ratio between the yield of ^225^Ac and the tracer ^243^Am was (with 0.8) in a similar range (literature value 1.1 (Cusnir et al. 2022), which is an important perquisite for the method.

Next, the separation (i.e. steps 3–5) was examined, using DGA branched resin for the solid-phase extraction. This material is, in contrast to the Y-imprinted resin from Cusnir et al. (2022), commercially available and typically used for the separation of actinides (Maxwell 2007; Vajda et al. 2020). The test started with a 3.5 M HNO_3_ solution, as this represented the actual solution after the microwave digestion (step 2), and 170 mBq ^225^Ac and 210 Bq ^243^Am were added. The washing and elution steps on the DGA branched columns were performed similar to the reported procedures for Am purification using the DGA normal resin (Maxwell 2007; Luisier et al. 2009; Vajda et al. 2020). 5 mL 4 M HNO_3_ were used twice for washing, and 15 mL 0.25 M HCl for elution of Am and Ac. The following steps were identical as previously described. The results showed a yield of only ~ 3% for ^225^Ac, which was significantly lower than the reported value from Cusnir et al. (2022). Also, the ^225^Ac/^243^Am yield ratio of 0.15 obtained was lower than the reported value of 0.9 (Cusnir et al. 2022). A reason for this could be the use of the DGA-branched resin.

Last, the complete analysis (i.e. steps 1 to 5) was examined, starting with 0.5 L of synthetic urine, which was spiked with 210 mBq ^225^Ac and 210 mBq ^243^Am. The coprecipitation was performed at pH 9 with CaPO_4_, which is commonly used for ^241^Am excretion analysis in the occupational monitoring laboratory of department MB 5 of the Federal Office of Radiation Protection, Germany. Afterwards, the precipitate was treated with 5 mL 14.5 M HNO_3_ and 1 mL 30% (v/v) H_2_O_2_ and placed in a microwave digestion system for 20 min. The resulting solution was filled up to 20 mL with dH_2_O, resulting in an approximately 3–4 M HNO_3_ solution. From that point on the treatment was the same as described before. The results showed, as expected from the previous experiment, a low ^225^Ac yield of < 1%. In comparison to the results reported in the literature (Cusnir et al. 2022), neither the yield nor the ^225^Ac/^243^Am ratio were similar. It is noteworthy that the yield for the tracer ^243^Am was only 1% in this test and therefore not well suited for a comparison or for drawing firm conclusions. Even though only one test for each step could be performed, the overall poor yields and - in case of the separation and complete analysis - also poor ^225^Ac/^243^Am ratio had to be addressed before a pilot phase could be initiated.

Due to the currently high demand of ^225^Ac and a resulting shortage of AcCl_3_ solution on the market, it was decided to continue the optimization of the method without the addition of ^225^Ac.

In order to further optimise the method, the electrodeposition (steps 4–5) was further examined. For this, the pH value was changed from 1.9 to 2.4, which is typically used by the authors for ^241^Am excretion analysis. First attempts did not significantly increase the ^243^Am yield. Therefore, the protocol of electrolysis sample preparation was modified by adding 0.6 mL 1 M NaHSO_4_ to the initial solution containing 15 mL 0.25 M HCl. Following the addition of 0.4 mL H_2_SO_4_ 18.2 M as the buffer system, the sample was evaporated to dryness, and the residue was calcinated for 30 to 60 min. Then, the residue was solved in 5 mL dH_2_O, transferred to the electrolysis cell and the beaker was rinsed with 5 mL dH_2_O, which was also transferred to the cell. Finally, the pH was adjusted to 2.4. The results (Table 1) showed an increase in ^243^Am yield of ~ 25% compared to the first test (electrodeposition: corresponding value for ^243^Am was 58.0%). Also, the yield was closer to the literature value of ~ 95%, indicating that the modified sample preparation protocol in combination with pH change from 1.9 to 2.4 prior to electrolysis had an important influence. From this point on, this electrodeposition protocol was used.

Next, the separation (steps 3–5) was performed according to the first test, with the modified electrodeposition. The results (Table 1) now showed a much higher ^243^Am yield with about 77% compared to the first test (separation: corresponding value for ^243^Am was 23.0%). Furthermore, the yield was in the same range compared to the literature value of ~ 73% (Cusnir et al. 2022). This showed that the chromatography using the DGA-branched resin worked, with the eluate treatment afterwards and electrolysis being the crucial part.

Finally, modified separation and electrodeposition steps were applied the complete extraction analysis was examined (steps 1–5). Compared to the first test, the only change made prior to the separation and following steps was adding 2 mL 6 M H_3_PO_4_ instead of 2 mL 14.8 M H_3_PO_4_ for CaPO_4_ precipitation. The 6 M H_3_PO_4_ solution was sufficient for a quantitative precipitation. This was checked by using 0.5 L of synthetic urine, adding 3 Bq ^241^Am, performing the precipitation with 6 M H_3_PO_4_, decanting the precipitate from the supernatant and measuring 0.2 L of the supernatant on a gamma detector (similar to the first gamma spectrometry tests) for any activity of ^241^Am. The results showed no ^241^Am signal, suggesting that the precipitation was rather complete (considering the detection limit of the measurement: with a detection limit of 0.05 Bq, the yield was estimated to be > 96% yield). Using the adjusted precipitation with the in the same protocol, the final result for the complete analysis (Table 1) showed with about 34% a much higher and more reliable ^243^Am yield compared to the first test with only 1% (which was the corresponding value for ^243^Am, for the complete procedure). Still, the ^243^Am yield reported in the literature (Cusnir et al. 2022) with ~ 60% was higher than the present value. Nonetheless, a tracer yield higher than 30% was considered enough for initiating a pilot phase and testing the developed ^225^Ac excretion analysis method by monitoring actual internal exposure, with the assumption that the Ac^3+^ and Am^3+^ ions behave similar during the radiochemical procedure.

**Table 1:** Comparison of ^243^Am yields obtained during different steps of the modified excretion analysis protocol with those from Cusnir et al. (2022)

Beginning step during radiochemical analysis	Electrodeposition	Separation	Complete
Radiochemical yield ^243^Am, preliminary tests [%]	**82.6 ± 12.3** *n* = 8	**76.8 ± 4.0** *n* = 6	**33.7 ± 10.9** *n* = 7
Radiochemical yield ^243^Am, literature [%]*	**95.4 ± 2.7** *n* = 10	**72.7 ± 15.2** *n* = 6	**59.7 ± 21.2** *n* = 18

* Literature values were calculated as mean values from the corresponding Table 1 (Electrodeposition), 2 (Separation) and 3 (Complete) (Cusnir et al. 2022), with the uncertainty presented here being the standard deviation of the values. n – number of samples or in case of literature given values used for mean value and standard deviation calculation

### Pilot phase of actinium-225 monitoring of internal exposure

The pilot phase was performed in cooperation with ITM with four of its employees (E1-E4), directly or indirectly involved in ^225^Ac production and usage. The aim was that each employee should collect a 24-hour urine sample every two weeks for six months during handling ^225^Ac, resulting in 12 samples per person and 48 samples in total. The samples should then be analysed with the developed method as described above. However, only 4–6 samples per individual could be collected, delivered and measured, due to operational reasons.

The samples were collected on any day between Friday and Monday by the employees, sent to the laboratory Tuesday afternoon and delivered with a parcel service provider on Wednesday morning. Upon arrival, the samples were registered, acidified for stability and then analysed with the above described method. The total processing time was two days, so that Thursday evening/Friday morning the alpha spectrometry results were obtained.

**Fig. 1:**
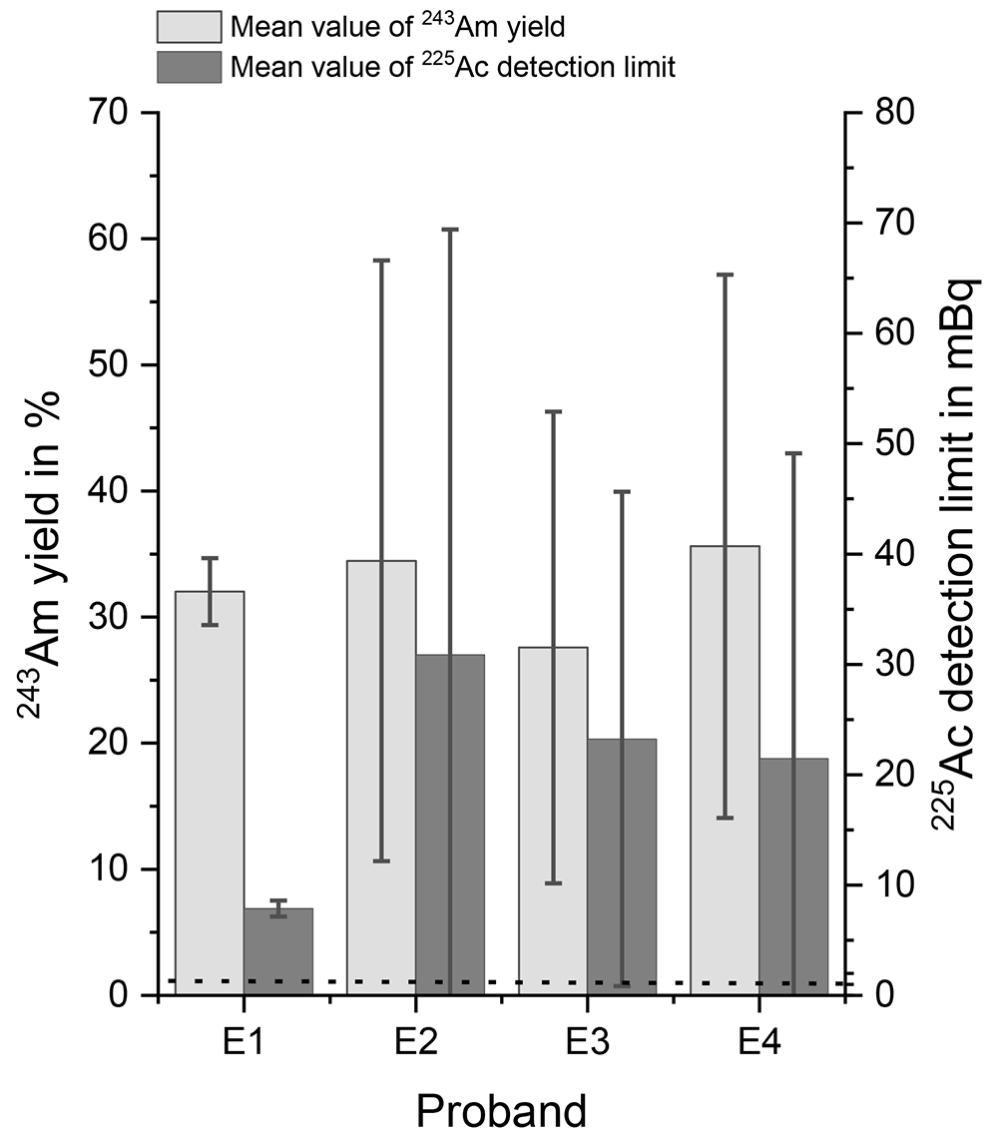
Results of the ^225^Ac excretion analysis pilot phase of monitoring four employees (E1-E4) for potential ^225^Ac intake. Dotted line represents the desired 1 mBq ^225^Ac detection limit

The ^243^Am yield mean value, the mean value of the ^225^Ac detection limit and the amount of samples s were: E1: 32.0% ± 2.7%, 7.9 ± 0.7 mBq (s = 4); E2: 34.5% ± 23.8%, 30.9 ± 38.5 mBq (s = 5); E3: 27.6% ± 18.7%, 23.2 ± 22.4 mBq (s = 5) and E4: 35.6% ± 21.5%, 21.5 ± 27.7 mBq (s = 6) (see also Fig. 1).

For neither of the four employees ^225^Ac signals were measured in the alpha spectrometry, with the ^243^Am yield being at least 27% (Fig. 1). The average ^243^Am yield was 32%, consistent with the previous findings (Table 1). The developed procedure could be integrated into the routine of laboratory work, and with the reagents and materials used, it did not pose a substantial challenge. Unfortunately, the obtained detection limits for ^225^Ac were higher than the desired value of 1 mBq for all samples. Furthermore, it must be considered that only four employees collected samples every two weeks, with occasional interruptions. In practice, monitoring for internal exposure due to ^225^Ac would require collection, shipment and processing of one sample per employee every week. Thus, laboratory capacities must be carefully evaluated in advance.

As quality control during the pilot phase, three synthetic urine samples were spiked by ITM with a known activity of ^225^Ac. The shipment of these samples was identical to the 24-hour urine samples of the employees. Upon arrival, the samples were diluted with synthetic urine leading to eight 0.5 L QA samples (QA1-QA8) with an activity range of 1 to 10 Bq. Note that these activities were up to 10,000 times higher than those expected in real conditions (about 1 mBq).

**Fig. 2:**
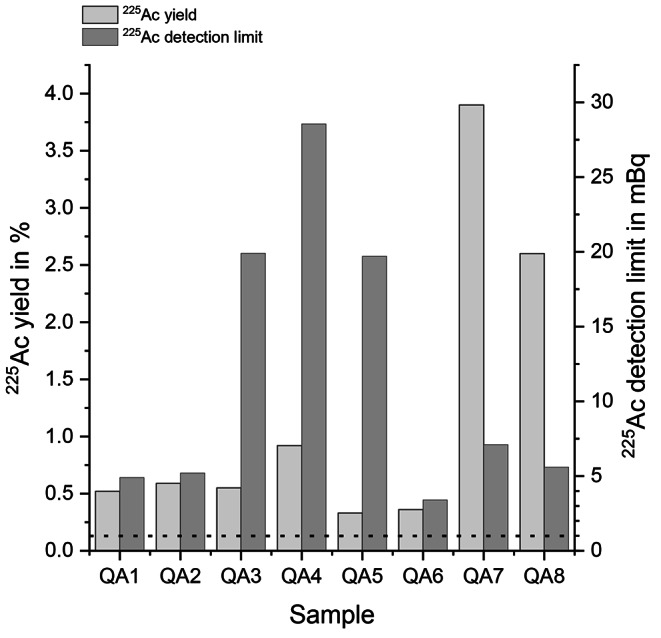
^225^Ac and ^243^Am yields of QA (quality assurance) samples during the pilot phase. Columns (light grey) represent ^225^Ac yield and (dark grey) ^225^Ac detection limit. Dotted line represents the desired 1 mBq ^225^Ac detection limit

The resulting ^225^Ac yields, ^243^Am yields, ^225^Ac/^243^Am yield ratios and ^225^Ac detection limit of the samples were: QA1: 0.5%, 52.3%, 0.01, 4.9 mBq; QA2: 0.6%, 49.8%, 0.01, 5.2 mBq; QA3: 0.6%, 62.4%, 0.01, 19.9 mBq; QA4: 0.9%, 39.0%, 0.02, 28.6 mBq; QA5 0.3%, 13.3%, 0.03, 19.7 mBq; QA6: 0.4%, 72.4%, 0.01, 3.4 mBq; QA7: 3.9%, 87.6%, 0.05, 7.1 mBq and QA8: 2.6%, 84.0%, 0.03, 5.6 mBq (see also Fig. 2).

The results (Fig. 2) showed, firstly, that it was possible to achieve ^243^Am yields of up to 84% with an average of 58%. This value was higher than in the preliminary tests and the measurement of the E1-E4 24-hour urine samples. The main reasons for this could be a better handling of the method over time, or that the human urine may be somewhat different from the synthetic urine. Second, the yield for ^225^Ac was between 0.3 and 3.9% with an average of 1.2%. The reasons for these poor ^225^Ac yields are yet to be determined. A possible explanation could be that the separation columns used are not as efficient as the Y-imprinted resin (Cusnir et al. 2022). These poor ^225^Ac yields also led to the third finding, that the ^225^Ac/^243^Am ratio was with an average of 0.02 50-times lower than the value of about 1 reported by Cusnir et al. (2022).

## Discussion

The aim of the present study was to develop a method that can be widely used for an excretion analysis of occupational internal exposure to ^225^Ac. Important factors for ^225^Ac excretion analyses are: a relatively short analysis, a detection limit lower than 1 mBq (based on the annual dose limit of 1 mSv for occupationally exposed persons (ISO 20553 2006), an ^225^Ac yield or tracer yield in case of AS greater than 50% (Vogl 1992), and an ^225^Ac/^243^Am yield ratio close to 1. The results obtained (Table 2) show that neither GS nor LSC are applicable for this purpose. While showing the best ^225^Ac yield (81%) and allowing for direct urine sample measurements, GS finally gave - even with measurements times up to 4 days - a detection limit that was higher than the required 1 mBq. Yet, this method can be used for special monitoring after incidents at workplace or for measurements of members of the public in emergency exposure situations. In special cases an effective dose of 1 mSv needs to be assessed from a single incident (quote translated: “in contrast to 1 mSv in the course of a year as in routine monitoring” ((RiPhyKo 2007). In emergency exposure situations only, incorporated activities leading to effective doses of 100 mSv need to be detected (AKI 2023). In both scenarios, the sampling would be conducted close to the intake, leading to relatively high excretion rates (e.g. after three days the excretion rate would be 3.5 times that that after seven days) and, thus, to higher detectable activities.

With a short measurement time and a reasonable yield at lower ^225^Ac concentrations than what could be measured with GS, the LSC measurements appeared applicable for the purpose of monitoring of internal exposure. However, at ^225^Ac activities lower than 5.9 Bq the ^225^Ac yield dropped drastically. Furthermore, analyses included dH_2_O and not urine samples. This could mean that the method finally would require an additional sample treatment prior to the activity measurements, resulting in a possible ^225^Ac loss during the process and in an increased time required for analysis. In addition, for LSC the detection limit was still higher than the required 1 mBq. In conclusion, also the LSC measurement is not helpful for routine occupational monitoring. However, similar to GS, LSC could be used for higher ^225^Ac activities to be detected. The third method examined here was the combination of solid-phase extraction in combination with AS. With an analysis duration of in general three days and a detection limit of 11 ± 8 mBq for the analysed QA samples, the method matched some of the requirements. However, even with a tracer yield of up to 87.6% the maximum ^225^Ac yield obtained was only 3.9% (see Fig. 2, sample QA7) in synthetic urine. The reasons for this disbalance in the ^225^Ac/^243^Am ratio, which Cusnir et al. (2022) had reported to be nearly 1 when they used their own method, could not addressed in the present study. Even with a better reliability of the method developed and described here with constantly high chemical yields, the reachable detection limits would not suffice to detect an annual effective dose of 1 mSv. However, a novel method has recently been developed that includes combined evaluation of several emissions of ^225^Ac and alpha spectra that are recorded throughout a year can significantly reduce the detectable dose to values even below 1 mSv (Meisenberg 2024).

**Table 2:** Comparison of Gamma spectrometry (GS), LSC measurements (LSC) and excretion analysis using alpha spectrometry (AS).

Method	Direct measurement^*^	Detection limit in a daily standard excretion [mBq]^§^	Measurement time [s]	Duration of analysis [d]	Lowest ^225^Ac activity analysed [Bq]^#^	^225^Ac yield [%]^†^	Detectable annual effective dose [mSv]^¶^
GS	Yes	4,900	80,000-320,000	1–4	8.9	81.2 ± 7.9	1.1·10^4^
LSC	Maybe	490	12,000	1^‡^	5.9	34.4 ± 23.5	1.1·10^3^
AS	No	11 ± 8	20,000	3	4.7	1.6 ± 1.4	26

* Referring to if potential 24-hour urine samples can be measured without any preparation prior to the activity measurement. For LSC measurements using the Hidex 300SL a method can be used to measure urine samples directly but this was not tested in the present study

§ The detection limit was calculated referring to the sample volume used for each method, i.e. 200 mL for GS, 20 mL for LSC and 500 mL for AS. The values presented for GS are mean values for all GS samples (i.e. GS1-GS6); LSC refer to sample LSC5; and AS are mean values for all QA samples (i.e. QA1-QA8, see Fig. 2)

‡ Since for LSC water-based samples were used, an analysis duration would be one day. If urine samples could not be measured directly, a sample treatment would be necessary and therefore longer durations could be expected

# The lowest ^225^Ac yield presented here is based, for LSC, on sample LSC5 since LSC6 had only 1% yield and the measured value was lower than the detection limit; for GS on sample GS4; for AS on the mean value for sample QA1, QA2, QA6, QA7 and QA8, since the other samples had ^225^Ac measured values lower than the corresponding detection limit

† ^225^Ac refers to the samples mentioned in footnote #

¶ Calculation according to ISO 20553 (2006), Eqs. 1 and 2 for a monitoring interval of 7 days

From an analytical point of view, the presented AS method is not optimal for monitoring of internal exposure of ^225^Ac, mainly due to the poor ^225^Ac/^243^Am yield ratio and the impossibility to reach the desired detection limit of 1 mBq. Furthermore, the results of the pilot phase presented here showed some disadvantages of the applied in vitro excretion analysis. The number of samples to be expected for a company working with ^225^Ac can be estimated based on the assumption that one has to take one 24-hour urine sample per week or 52 samples per year per employee who is in contact with ^225^Ac. This translates into a considerable workload and financial burden for the company. For those employees who finally have to collect a sample every week, this might pose a non-negligible challenge. During the pilot phase described here, although the employees were asked to collect the samples only every second week, the number of samples collected was only 50% of the “target” value.

## Conclusion

The study described here represents a first attempt to develop a method for the in vitro incorporation monitoring of ^225^Ac. Although the analytical methods of gamma spectrometry and liquid scintillation counting showed very good ^225^Ac yields, the required detection limit of 1 mBq could not be reached by several orders of magnitude. Nevertheless, both methods are suitable for analysing samples in emergency exposure situations that would presumably include a significantly higher ^225^Ac activity than the samples to be analysed in routine incorporation monitoring, partly because they allow for direct measurements.

The alpha spectrometry method developed based on the work of Cusnir et al. (2022) showed promising results. Detection limits close to 1 mBq were achieved, together with very good tracer yields. However, the ^225^Ac yields of around 2% were far too low. It is clear that intensive research is still required to bring the yields of ^225^Ac to the same level as those of the tracer ^243^Am.

It is concluded that methods other than those investigated in the present study, or the combination of different methods should be explored for the monitoring of internal exposure to ^225^Ac. This could involve air monitoring with an occasion-based in vivo whole-body measurement and in vitro excretion analysis of the employees as an additional feature. However, the exact details and procedures still need to be defined, especially in the area of room air monitoring, and decided by the competent authorities.

## Data Availability

No datasets were generated or analysed during the current study.
